# Composition and temperature-dependent phase transition in miscible Mo_1−*x*_W_*x*_Te_2_ single crystals

**DOI:** 10.1038/srep44587

**Published:** 2017-03-15

**Authors:** Yang-Yang Lv, Lin Cao, Xiao Li, Bin-Bin Zhang, Kang Wang, B P Bin Pang, Ligang Ma, Dajun Lin, Shu-Hua Yao, Jian Zhou, Y. B. Chen, Song-Tao Dong, Wenchao Liu, Ming-Hui Lu, Yulin Chen, Yan-Feng Chen

**Affiliations:** 1 National Laboratory of Solid State Microstructures & Department of Materials Science and Engineering, Nanjing University, Nanjing 210093 China; 2 National Laboratory of Solid State Microstructures & Department of Physics, Nanjing University, Nanjing 210093 China; 3 Institute of materials Science and Engineering, Jiangsu University of Science and Technology, Zhenjiang 212003 China; 4 Institute of Advanced Materials (IAM) & Jiangsu National Synergetic Innovation Center for Advanced Materials (SICAM), Nanjing Tech University, Nanjing 211800 China; 5 School of Physical Science and Technology, Shanghai Tech University, Shanghai 200031, China; 6 State Key Laboratory of Low Dimensional Quantum Physics, Collaborative Innovation Center of Quantum Matter and Department of Physics, Tsinghua University, Beijing 100084, China; 7 Collaborative Innovation Center of Advanced Microstructure, Nanjing University, Nanjing, 210093 China

## Abstract

Transition metal dichalcogenides (TMDs) WTe_2_ and MoTe_2_ with orthorhombic Td phase, being potential candidates as type-II Weyl semimetals, are attracted much attention recently. Here we synthesized a series of miscible Mo_1−*x*_W_*x*_Te_2_ single crystals by bromine vapor transport method. Composition-dependent X-ray diffraction and Raman spectroscopy, as well as composition and temperature-dependent resistivity prove that the tunable crystal structure (from hexagonal (2H), monoclinic (β) to orthorhombic (Td) phase) can be realized by increasing W content in Mo_1−*x*_W_*x*_Te_2_. Simultaneously the electrical property gradually evolves from semiconductor to semimetal behavior. Temperature-dependent Raman spectroscopy proves that temperature also can induce the structural phase transition from β to Td phase in Mo_1−*x*_W_*x*_Te_2_ crystals. Based on aforementioned characterizations, we map out the temperature and composition dependent phase diagram of Mo_1−*x*_W_*x*_Te_2_ system. In addition, a series of electrical parameters, such as carrier type, carrier concentration and mobility, have also been presented. This work offers a scheme to accurately control structural phase in Mo_1−*x*_W_*x*_Te_2_ system, which can be used to explore type-II Weyl semimetal, as well as temperature/composition controlled topological phase transition therein.

Recently, layered transition metal dichalcogenides (TMDs) materials have attracted extensive attention because of their superior properties, for example, large thermoelectric effect in TiSe_2_ at room temperature^1^, superconductivity^2^, charge density waves^3^, extremely large magnetoresistance in WTe_2_^4^, topological phase^5^,^6^ and next-generation (opto-) electronics devices^7^,^8^. Among these transition metal dichalcogenides, hereafter we focus on Mo_1−*x*_W_*x*_ (Te, Se, S)_2_ compounds.

Mo_1−*x*_W_*x*_(S, Se, Te)_2_ compounds demonstrate a rich crystal structures and diversified physical properties^9^,^10^,^11^,^12^. In the viewpoint of crystal structure, Mo_1−*x*_W_*x*_ (Te, Se, S)_2_ can crystallize into three phases under different experimental conditions, including 2H- (hexagonal, space group *P63/mmc*), Td- (orthorhombic, space group *Pmn2*_*1*_) and β-phase (monoclinic, space group *P2*_*1*_/*m*), as shown in Fig. 1(a)^13^. The common structure of MoTe_2_ is 2H-phase, while the WTe_2_ is normally taken Td-phase. The β-phase MoTe_2_, a metastable phase, can also be obtained by quenching method at high temperature of about 1173 K^14^,^15^. It has the inversion symmetry which does not in Td phase. In addition, these phases may be changed under thermal agitation, for example, MoTe_2_ has a first-order structural phase transition (around 250 K) from the β to the Td polytype^15^,^16^. In the viewpoint of physical properties, generally speaking, the 2H phase is a semiconductor, but Td and β phase are semi-metallic. For example, 2H-phase Mo_1−*x*_W_*x*_S_2_ and Mo_1−*x*_W_*x*_Se_2_ are semiconductors whose electronic band gap can be continuously tuned by alloy^9^,^10^,^11^,^12^. While, MoTe_2_ compounds with β and Td phase show the metallic conductivity. It should be emphasized that both MoTe_2_ and WTe_2_ with Td phase belong to a type-II topological Weyl semimetal according to recent theoretical predictions^17^,^18^. The condensed matter physics counterparts of Weyl fermions have been realized firstly in type-I Weyl semimetals in the TaAs family^19^,^20^,^21^,^22^. Different from type-I Weyl semimetal, Dirac cone in type-II Weyl semimetal is tilted^17^,^18^. Currently, several angle-resolved photoemission spectroscopy and transport works have claimed to observe the Fermi arc and tilted Dirac cones in type-II Weyl semimetals of WTe_2_ and MoTe_2_^23^,^24^,^25^,^26^,^27^,^28^. In addition, Mo_1−*x*_W_*x*_Te_2_ can demonstrate the topological phase transition under thermal agitation or alloy. For example, the length of Fermi arc in Td phase WTe_2_ is tunable by temperatures or by Mo-alloy as theoretically predicted^29^,^30^.

Here we summarized the previous effort to elucidate the phase transition in Mo_1−*x*_W_*x*_Te_2_. For β-MoTe_2_, there is a phase transition from the β to the Td polytype at about 250 K in β-MoTe_2_^15^,^16^. It is worthwhile to mention that the Td-phase is an important candidate to materialize the newly predicted type-II Weyl semimetal. Type-II Weyl semimetals have a series of novel physical properties, such as anisotropic negative magentoresistance, topological anomalous Hall effect^17^,^18^. The transition from β- to Td-phase is also characterized by the temperature-dependent XRD^31^,^32^, as well as temperature-dependent Raman spectroscopy^33^,^34^. However, the existence of orthorhombic Td phase MoTe_2_ is still under hot debate because it is quite challenging to directly distinguish the subtle differences between the Td and β phase. As for the Mo_1−*x*_W_*x*_Te_2_ system, there are some experimental studies on ceramic samples^35^,^36^ and theoretical predictions on monolayers^37^,^38^, but no any phase-transitions works for single crystal samples at present, to the best of our knowledge.

Based on above-mentioned discussions, it is quite crucial to map out the phase diagram of Mo_1−*x*_W_*x*_Te_2_ at different temperature and their corresponding electrical properties in order to explore the type-II topological Weyl semimetals and corresponding novel physical properties in this system. Here, we synthesized a series of Mo_1−*x*_W_*x*_Te_2_ single crystals by the chemical vapour transport method. X-ray diffraction and chemical composition analysis confirm that the obtained samples have single crystalline quality, in which the Mo and W elements are miscible. The composition-dependent and temperature-dependent phase evolutions in Mo_1−*x*_W_*x*_Te_2_ are determined by Raman spectroscopic characterization. These characterizations substantiate that by increasing the W composition, the crystal structure of Mo_1−*x*_W_*x*_Te_2_ gradually changes from 2H, β to Td phase^13^. Simultaneously, the electrical properties gradually evolve from semiconductor to semimetal behavior revealed by temperature-dependent resistivity and Hall curves. Based on these data, we also map out the composition- and temperature-dependent phase diagram of Mo_1−*x*_W_*x*_Te_2_ system.

## Methods

### Crystal Growth

**Figure 1:**
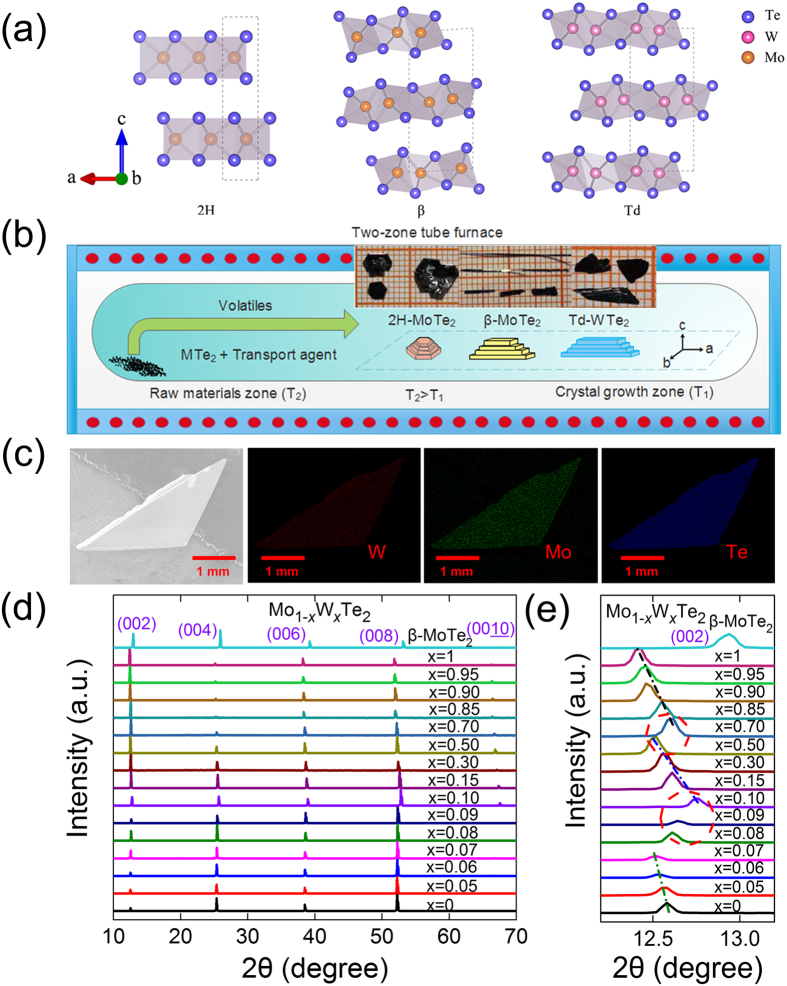
(**a**) Crystal structure of Mo/WTe_2_ (2H, β and Td phase, respectively). (**b**) Schematic of the double-zone CVT growth furnace and the growth process of Mo_1−*x*_W_*x*_Te_2_ single crystals. (**c**) SEM image and W (red), Mo (green) and Te (blue) element mapping images of Mo_0.5_W_0.5_Te_2_ single crystal. (**d**) The XRD patterns of representative Mo_1−*x*_W_*x*_Te_2_ single crystals. (**e**) The enlarged (002) XRD peaks for all samples.

A series of Mo_1−*x*_W_*x*_Te_2_ single crystals were prepared by the chemical vapor transport (CVT) method that is discussed in detail elsewhere^39^. Figure 1(b) shows a schematic of the double-zone CVT growth furnace with well-controlled temperature. The single-crystal growth procedure includes two steps. Firstly, all Mo_1−*x*_W_*x*_Te_2_ polycrystalline samples were synthesized by heating a stoichiometric mixture of high purity elemental powders W (Alfa Aesar 99.99%), Mo (Alfa Aesar 99.99%) and Te (Alfa Aesar 99.999%) by solid state reaction at 1073 K in evacuated quartz tubes. Secondly, Mo_1−*x*_W_*x*_Te_2_ crystals were grown by CVT method using Br_2_ (about 5 mg/mL) as transport agent in the sealed evacuated quartz tube at a double zone furnace. By means of optimized the growth parameters, large size (centimetre-level) and high-quality crystals with regular shape can be obtained. The β-MoTe_2_ crystals can be grown at high temperature profile of 1173~1273 K. The growth quartz tube was quenched in ice water to yield the high-temperature β phase. And 2H-MoTe_2_ and Td-WTe_2_ crystals were obtained with a temperature profile of 1023~1123 K using usual cooling treatment (100 K/h) without quenching.

### Material Characterization

The elemental compositions of the samples were determined by energy dispersive X-ray spectroscopy (EDS) analysis conducted on an FEI Quanta 200 FEG environmental scanning electron microscope (SEM). X-ray diffraction (XRD) measurements were performed on the crystals using an X-ray diffractometer (Ultima III Rigaku, Cu-*K*_α_ radiation as an X-ray source). The scanning rate of 3° per minute and 2θ scanned from 10° to 70° were used to collect XRD data. Raman spectra were taken by a backscattering geometry on a LabRam HR800 Microscope system (Horiba Jobin Yvon), using the 633 nm a He-Ne laser as an optical source. Standard four-probe technique was used for resistivity and Hall-effect measurements on a Quantum Design PPMS-9.

### The density functional theory (DFT) Calculation

The Raman frequencies of MoTe_2_ and WTe_2_ for different structures were calculated by DFT in the generalized gradient approximation implemented in the Vienna Ab-initio Simulation Package (VASP) code^40^,^41^ and the Phonopy software^42^. The projected augmented wave method^43^,^44^ and the van der Waals corrected optB86b-vdw functional^45^,^46^ are used. The plane-wave cutoff energy is 500 eV throughout the calculations. The *k* point mesh is 12 × 12 × 4 for the 2H phase and 8 × 14 × 4 for the β- and Td-phase. The atom positions and lattice constants are optimized until the maximal residual force is less than 0.002 eV/Å. The optimized lattice constants are very well consistent with the reported values which are shown in Table S1 at Supplementary Information.

## Results and Discussion

Figure 1(c) depicts the SEM images of the as-grown Mo_0.5_W_0.5_Te_2_ crystals and the corresponding EDS mapping of Mo, W and Te elements, respectively. As can be seen, the three elements are uniformly distributed in the sample, strongly suggesting the growth is homogeneous. The composition analysis substantiates that the composition ratio between Mo and W in this sample is 1.00:1.02, which is in agreement to the designed chemical compositions. All the EDS spectra of the as-grown Mo_1−*x*_W_*x*_Te_2_ crystals are depicted in Fig. S1. The elemental compositions of all the crystal samples and cross section used in EDS analysis are shown in Table II and III in Supplementary Information. The XRD patterns of single crystal samples are presented in Fig. 1(d). All peaks indexed as the (0 0 2*k*) reflections, indicating that the exposed surfaces of the crystals belong to *c*-plane. The full-width at half maximum of Mo_1−*x*_W_*x*_Te_2_ (002 pole) series samples varies from 0.07° to 0.09°, which infers the as-grown single crystals have high crystalline quality. In order to show the effect of isovalent substitution on the crystal structure clearly, we enlarge of the series (002) peaks in Fig. 1(e) for all Mo_1−*x*_W_*x*_Te_2_ samples. It is evident that there are three continuous change regimes (*x* = 0~0.07, 0.10~0.50, and 0.70~1, respectively), which implies there may be three different phases. This may be due to the different lattice parameter *c* of 2H-MoTe_2_, β-MoTe_2_ and Td-WTe_2_ (13.97, 13.86, and 14.07 Å, respectively) that causes the angle-shift of the (002)-peaks^13^. In addition, we also can see that the diffraction peaks gradually shift to lower angle degree with increasing *x* within each concentration range. This is because that the lattice parameter *c* changes large due to the ionic radius of W^4+^ (0.66 Å) being larger than that of Mo^4+^ ions (0.65 Å)^39^,^47^. But, by careful inspection, we find that from *x* = 0.08 to 0.10 and from *x* = 0.50 to 0.70, the diffraction (002) peaks gradually shift to higher angle degree with increasing *x* (W composition). The estimated *c*-axis lattice parameter *d*_*c*_ as a function of *x* for Mo_1−*x*_W_*x*_Te_2_ system and β-MoTe_2_ is given as Fig. S2 at Supplementary Information. So it may be reasonable to conclude that the phase transitions occur from *x* = 0.08 to 0.1 and from *x* = 0.50 to 0.70. Crystal structures of Mo_1−*x*_W_*x*_Te_2_ compounds change from 2H, β to Td phase with increasing *x*.

**Figure 2:**
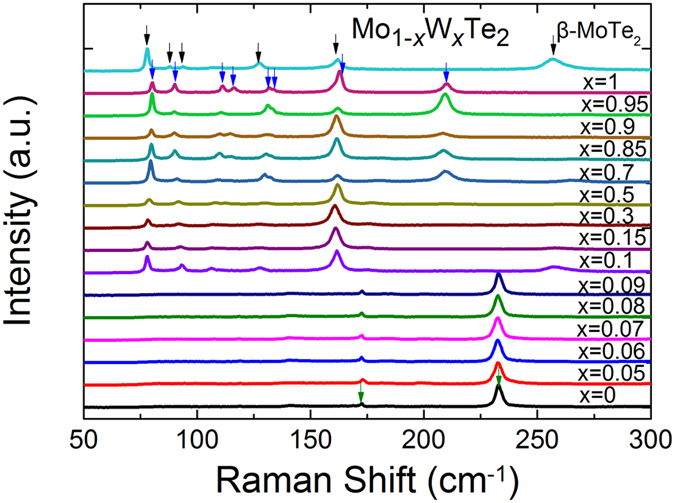
Unpolarized Raman spectra on the *ab* plane of Mo_1−*x*_W_*x*_Te_2_ single crystals measured at room temperature.

In order to substantiate the composition-dependent structure phase transitions, Raman spectra measurements were further used to characterize these single crystals at room temperature. Figure 2 shows the normalized un-polarized Raman spectra from the *ab* plane of the as-grown Mo_1−*x*_W_*x*_Te_2_ single crystalline samples and the Raman spectrum of pure β-MoTe_2_ obtained from high temperature quenching (upmost in Fig. 2). Obviously, there are three different types of Raman spectra mapped to different *x* range (0~0.09, 0.10~0.50, and 0.70~1, respectively) and the Raman spectrum of β-MoTe_2_ (upmost) is in good agreement with that of *x* from 0.1 to 0.50. These general trends are in agreement to XRD results.

To understand the Raman modes of different crystalline phases in the Mo_1−*x*_W_*x*_Te_2_ system, we also calculated the Raman frequencies by the DFT and the results are well consistent with the experimental one (see Fig. S3 at Supplementary Information). According to the group theory analysis, the irreducible representations of the phonons in bulk 2H-MoTe_2_ (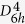 point group) at the center (Γ point) of the Brillouin zone (BZ) are 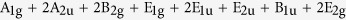, where E_2g_ (24.993 and 230.043 cm^−1^), E_1g_ (116.540 cm^−1^), A_1g_ (171.893 cm^−1^) are Raman-active. In bulk β-MoTe_2_ or WTe_2_ (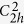), the calculated phonon modes at the Γ point include 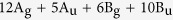, where there are 18 Raman active phonon modes (12A_g_ + 6B_g_), as shown in Fig. S3. Bulk Td-MoTe_2_ and Td-WTe_2_ both belong to the 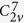 point group, the group theory analysis indicates that the BZ-center vibration modes decompose into 36 one-dimensional irreducible representations: 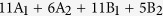, where all modes are Raman active (see Fig. S3). All the above calculation results are in agreement with the previous theoretical works^48^,^49^,^50^.

To analyse the Raman results of Mo_1−*x*_W_*x*_Te_2_ compounds, the Raman results of some pure phases are mentioned here firstly. As depicted in Fig. 2, in Raman spectra measurements of 2H-MoTe_2_ (the bottom black line), we detect two sharp peaks at 172.8 and 232.9 cm^−1^ which are attributed to the A_1g_ and E_2g_ modes, respectively. For β-MoTe_2_ crystals (see upmost line of Fig. 2), we observe six Raman peaks at around 78.0, 88.0, 94.0, 127.4, 162.2 and 256.8 cm^−1^, associated with the A_g_ and B_g_ modes, respectively. And in the Raman spectrum of Td-WTe_2_ (the second line from the top in Fig. 2), there are eight peaks centred at 80.2, 90.0, 111.3, 116.2, 131.7, 133.5, 162.8 and 210.3 cm^−1^, respectively, which are ascribed to the A_1_ and A_2_ modes. These experimental Raman frequencies are very well reproduced by our DFT calculations (see Fig. S3 in Supplementary Information).

**Figure 3:**
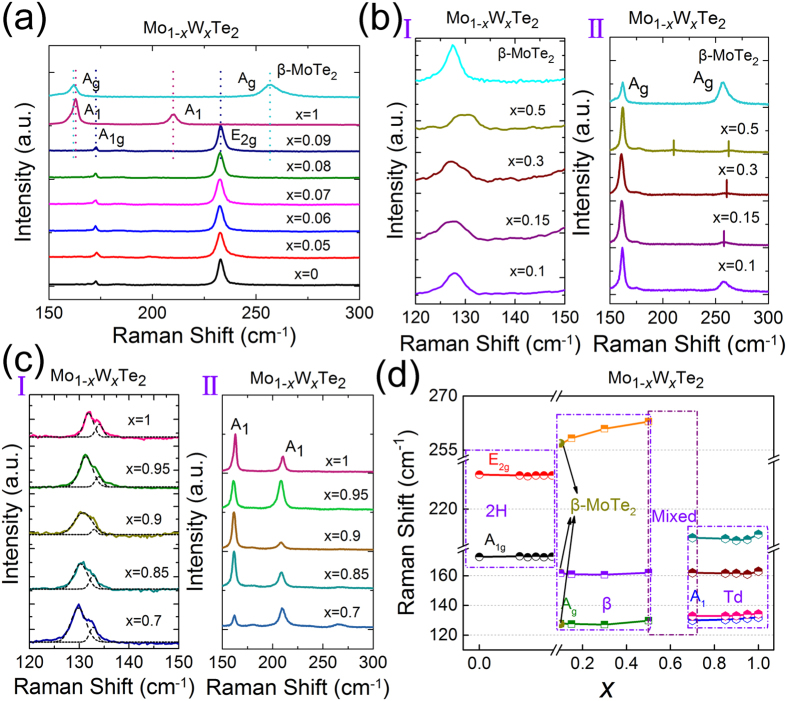
(**a**) Raman spectra (150~300 cm^−1^) of Mo_1−*x*_W_*x*_Te_2_ single crystals with composition *x* in the range of 0~0.09. (**b**) Raman spectra (I: 120~150 cm^−1^; II: 150~300 cm^−1^) of β-MoTe_2_ and Mo_1−*x*_W_*x*_Te_2_ single crystals with *x* composition in the range of 0.1~0.5. (**c**) Raman spectra (I: 120~150 cm^−1^; II: 150~300 cm^−1^) of Mo_1−*x*_W_*x*_Te_2_ single crystals with *x* composition in the range of 0.7~1.0. (**d**) Composition-dependent Raman frequencies of β-MoTe_2_ and Mo_1−*x*_W_*x*_Te_2_ single crystals (*x* = 0~1.0). All the Raman spectra were measured at room temperature.

Using the above-mentioned Raman data in the pure phases, we can track the composition-dependent structure evolution in the whole Mo_1−*x*_W_*x*_Te_2_ systems. The evolutions of the prominent Raman peaks for different *x* in three frequency-ranges are plotted in Fig. 3(a–c). And the corresponding shifts of these peaks as functions of *x* are summarized in Fig. 3(d), respectively. We compare the Raman active modes of the three phases, two prominent peaks, A_1g_ (near 171.893 cm^−1^) and E_2g_ (near 230.043 cm^−1^), in the 2H phase spectra are assigned as fingerprint peaks. Because there are no Raman active modes near the above two positions in β and Td phases. As shown in Fig. 3(a), from *x* = 0 to 0.09, two prominent peaks were observed at about 173 and 233 cm^−1^, respectively. These two peaks are related to the A_1g_ and E_2g_ modes of 2H-MoX_2_, confirming the corresponding samples being 2H phase. Figure 3(d) indicates that there is no noticeable frequency change of the two peaks with the *x* composition increased from 0 to 0.09. But the intensity and full-width-at half-maximum of A_1g_ and E_2g_ mode in 2H phase are irregularly dependent on W-concentration, whose mechanism will be explored in the near future. It also should be mentioned that according to a previous report^51^, the Raman spectrum was strongly dependent on electron/hole doping in *single layered* MoS_2_. In that case, Fermi level adjustment should affect electron/hole concentration, which will in turns affect the electron-phonon scattering and Raman peaks. Different from this scenario, iso-valence W-doping to Mo in our samples does not induce significantly the electron/hole concentration, therefore the peaks of A_1g_ and E_2g_ have not obviously changed as shown in Fig. 3(d).

According to the previous works^33^,^34^, the evolution of the Raman mode at near 130 cm^−1^ is a direct verification of the structural phase transition of MoTe_2_ from high temperature β to low temperature Td phase. In addition, there are different Raman signals for β- and Td-phase at the range of 150~300 cm^−1^. Here we used peaks from 120 to 300 cm^−1^ to determine the β-Td phase transition. The Raman peaks around 130 cm^−1^, and those from 150 to 300 cm^−1^ are enlarged in Fig. 3(b) and (c) for β-MoTe_2_ and the Mo_1−*x*_W_*x*_Te_2_ (*x* = 0.1~1.0), respectively. We can see that from *x* = 0.1 to 0.5 there is only one peak found near 127 cm^−1^ in the Raman spectra between 120~150 cm^−1^, as well as β-MoTe_2_ (see Fig. 3(b-I)). The position of this peak exhibits slight red-shift compared with that of β-MoTe_2_ (see Fig. 3(d)). There are also two peaks found at around 162 and 257 cm^−1^ in the Raman spectra of Mo_1−*x*_W_*x*_Te_2_ crystals (*x* = 0.1~0.5) (see Fig. 3(b-II)), which agree to the Raman signal (A_g_ modes) of β-MoTe_2_. From Fig. 3(d), it can be seen that the positions of the peaks (162 and 257 cm^−1^) exhibit slight blue-shift compared with that of β-MoTe_2_. The above results infer that these samples within *x* = 0.1~0.5 may belong to β-phase. On the other hand, when *x* changed into the range of 0.7–1.0, we find a multiple peak at around 130 cm^−1^ in the Raman spectra between 120~150 cm^−1^ (shown in Fig. 3(c-I)). The whole peak is fitted by the Lorentz function. As shown in Fig. 3(c-I), the experimental peaks can be fitted with two Lorentz line shapes with central peaks at approximately 130 and 133 cm^−1^, respectively. It suggests that the compound structure change from β to Td phase and the Mo_1−*x*_W_*x*_Te_2_ samples with *x* ranged from 0.7 to 1.0 have Td structure. Compared with pure Td-WTe_2_, the positions of the two peaks (near 130 and 133 cm^−1^) in all other samples exhibit red-shift and the difference is increased gradually with Mo composition increased (see Fig. 3(d)). In addition, from *x* = 0.7 to 1, we detect another two peaks at around 162 and 210 cm^−1^ in the Raman spectra measurements (shown in Fig. 3(c)-II), confirming all the Mo_1−*x*_W_*x*_Te_2_ samples (*x* = 0.7~1) belong to Td phase too. The positions of the two peaks in all other samples exhibit red-shift compared to pure Td-WTe_2_ (see Fig. 3(d)).

It is worth mentioning the Raman spectra of Mo_0.7_W_0.3_Te_2_, Mo_0.5_W_0.5_Te_2_ and M_0.3_W_0.7_Te_2_ are enlarged in Fig. S4. In Mo_0.5_W_0.5_Te_2_, except the characteristic peaks of β phase, there is a very weak peak around 210 cm^−1^, which is likely to be related to the Raman signal of the A_1_ modes of Td phase^33^. And broad Raman peak at around 130 cm^−1^ of Mo_0.5_W_0.5_Te_2_ also could be approximately as overlap of multiple peaks. These results indicate that Mo_0.5_W_0.5_Te_2_ have mixture of β- and Td-phase. In addition, a low-intensity peak around 264 cm^−1^ is observed in the Mo_0.3_W_0.7_Te_2_ Raman spectrum, which is contributed to the Raman signal of the A_g_ modes in β-MoTe_2_. It suggests that around *x* = 0.7, the phase can be approximately changed into β phase. Based on above-mentioned analysis, we conclude that the samples with *x* range of 0~0.09, 0.10~0.50, and 0.70~1, belong to 2H-, β- and Td phase, respectively. And at the range of *x* = 0.5~0.7, the phase of Mo_1−*x*_W_*x*_Te_2_ can be ascribed to be a mixing phase of β and Td. The critical compositions of phase transition in the Mo_1−*x*_W_*x*_Te_2_ system are approximately located at around *x* = 0.1 and 0.5, respectively.

**Figure 4:**
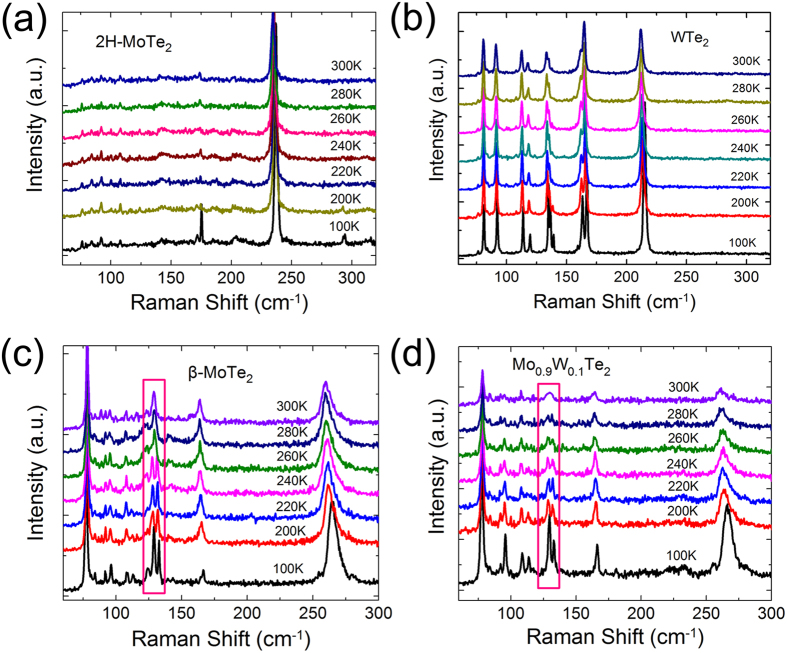
(**a**) Temperature-dependent Raman spectra of 2H-MoTe_2_ crystals. (**b**) Temperature-dependent Raman spectra of Td-WTe_2_ crystals. (**c**) Temperature-dependent Raman spectra of β-MoTe_2_ crystals. (**d**) Temperature dependence Raman spectra of Mo_0.9_W_0.1_Te_2_ (129 cm^−1^) and Mo_0.5_W_0.5_Te_2_ (130 cm^−1^) samples.

Except the composition-dependent structure phase transition, we further characterized the temperature-dependent structure phase transition in Mo_1−*x*_W_*x*_Te_2_ compounds. As shown in Fig. 4(a) and (b), with decreasing the temperature of 2H-MoTe_2_ and Td-WTe_2_ samples from 300 to 100 K, no new Raman peaks appear although all peaks exhibit different blue-shift. This result implies that no temperature phase transition occurs in 2H-MoTe_2_ and Td-WTe_2_ systems at low-temperature range under atmospheric pressure. In Fig. 4(c), we present temperature-dependent Raman spectra for β-MoTe_2_ at the range between 100~300 K. One can see the intensity of all peaks is strengthened although the magnitude of the blue-shift is different. Surprisingly, at around 240 K, the Raman peaks of 129 cm^−1^ become two new peaks at about 127 and 132 cm^−1^, respectively, reaching the maximum intensity below 200 K, as highlighted in pink wireframe. In accordance with previous analysis^33^,^34^ and our calculation results, the two new Raman peaks occur only at the low temperature Td phase in the Mo_1−*x*_W_*x*_Te_2_ system. It suggests that splitting of this Raman peak may infer the structural phase transition in β-MoTe_2_. In order to verify the low-temperature phase transition, we also characterized the temperature dependence Raman spectra on Mo_0.9_W_0.1_Te_2_ of β phase. The evolution of the peak 129 cm^−1^ is shown in Fig. 4(d). Upon cooling, the peak develops into two new Raman peaks at about 280 K, confirming that the two sample change from high temperature β to low temperature Td phase.

**Figure 5:**
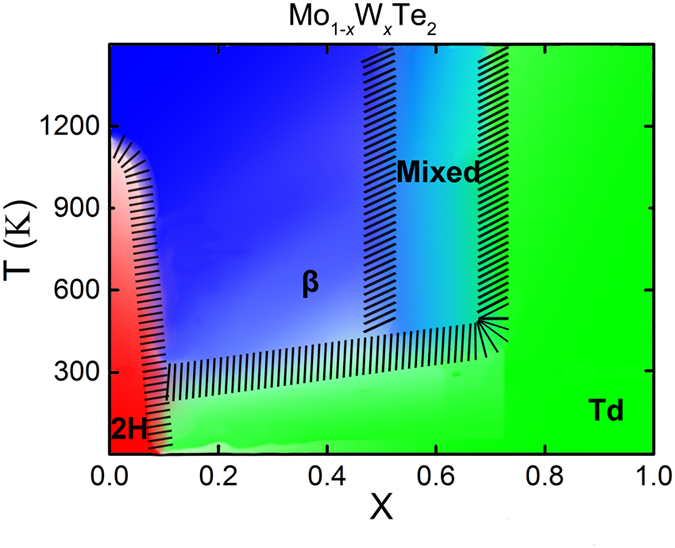
Phase diagram of Mo_1−*x*_W_*x*_Te_2_ system as functions of composition *x* and temperature. The areas, where the adjacent phases are coexisting, are shaded by black oblique line.

Based on the above analysis, we plot a structural phase diagram of Mo_1−*x*_W_*x*_Te_2_ as functions of composition *x* and the temperature in Fig. 5. It is evident that 2 H phase appears in a composition range from *x* = 0 to 0.09 at room temperature and pure 2H-MoTe_2_ transforms into the high-temperature phase β-MoTe_2_ at about 1173 K^14^,^15^. β phase exists in a composition range of *x* = 0.1~0.5 at room temperature and would change from β to low temperature Td phase at 240~300 K. Td phase is a candidate of type-II Weyl semimetal, so the corresponding phase transition can be designated as the temperature-induced topological phase transition. In the Mo_1−*x*_W_*x*_Te_2_ alloys, Td phase (a candidate of type-II Weyl semimetal) lies in a composition *x* range of 0.7~1, but they have no temperature-dependent phase transitions. In addition, at the range of *x* = 0.5~0.7, the phase of Mo_1−*x*_W_*x*_Te_2_ can be described as a mixing phase of β and Td.

**Figure 6:**
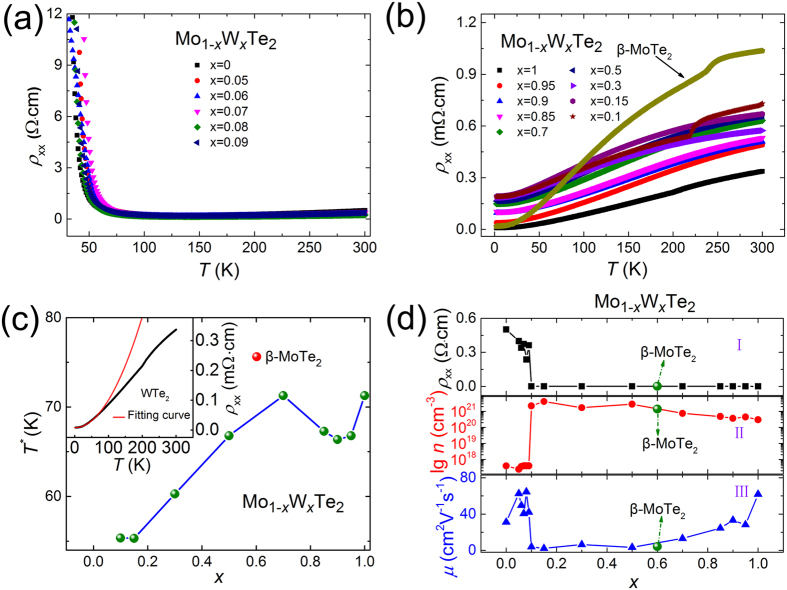
(**a**) Temperature-dependent *ab*-plane resistivities *ρ*_*xx*_ of Mo_1−*x*_W_*x*_Te_2_ single crystals (*x* = 0~0.09). (**b**) Temperature-dependent *ab*-plane resistivities *ρ*_*xx*_ of β-MoTe_2_ and Mo_1−*x*_W_*x*_Te_2_ single crystals (*x* = 0.1~1). (**c**) Composition-dependent of T* in Mo_1−*x*_W_*x*_Te_2_ single crystals (*x* = 0.1~1). Red globule represents β-MoTe_2_ samples. Upper inset shows temperature dependence *ab*-plane resistivity of Td-WTe_2_. Black symbols are the experimental data and the red line represents a fit with the Fermi liquid model 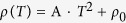. (**d**) Composition-dependent of the *ab*-plane resistivities (I), carrier concentration (II) and mobility (III) of Mo_1−*x*_W_*x*_Te_2_ single crystals measured at room temperature. Green globules represent β-MoTe_2_ samples.

The electrical properties of the Mo_1−*x*_W_*x*_Te_2_ compounds were also characterized. As shown in Fig. 6(a), from *x* = 0 to 0.09, the Mo_1−*x*_W_*x*_Te_2_ samples all show the semiconductor behavior. While the other Mo_1−*x*_W_*x*_Te_2_ samples with a composition range of *x* = 0.1~1 and β-MoTe_2_ samples show the semi-metallic behavior as presented in Fig. 6(b). Interestingly, for β-MoTe_2_ and Mo_0.9_W_0.1_Te_2_, obvious electrical resistivity anomalies are observed at 250 and 230 K respectively, which are associated with the structural phase transition from the β to Td phase. However, there are no resistivity anomaly appeared in the temperature-dependent-resistivity curves of Mo_1−*x*_W_*x*_Te_2_ samples (*x* = 0.15~0.50). In addition, the resistivity of Td-WTe_2_ below 71 K (see upper inset of Fig. 6(c)) can be well fitted by


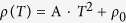


where *ρ*_0_ is the resistivity at 0 K and A is constant. It suggests that electrons in Td-WTe_2_ at low temperature can be well described by Landau Fermi liquid theory. The temperature dependent resistance of Td-WTe_2_ indeed shows a transition (T^*^) from linear behaviour originating from the electron-phonon coupling at high temperatures to the Landau Fermi liquid behaviour with dominant electron-electron scattering at low temperatures^52^. With the same method, we fitted the temperature-dependent resistivity of the other metallic phase samples. As shown in Fig. 6(c), it is found that upon raising the Mo concentration, T^*^ of these samples gradually decreases, compared with Td-WTe_2_. And the T^*^ of β-MoTe_2_ is 75 K. The calculation procedure of carrier concentrations and carrier mobilities is shown in Supplementary Information. Figure 6(d) summarized composition-dependent of the *ab*-plane resistivities (I), carrier concentration (II) and mobility (III) of Mo_1−*x*_W_*x*_Te_2_ single crystals measured at the room temperature. One can see, with increasing *x*, the *ab*-plane resistivity gradually decreases, the carrier concentration first increases and then rapidly decreases, while the mobility first decreases and then increases. Quantitatively, the *ab*-plane resistivities of 2H-MoTe_2_, β-MoTe_2_, and Td-WTe_2_ are 0.5, 1.0 × 10^−3^ and 3.4 × 10^−4^ Ω·cm, respectively. The carrier concentrations are 4.0 × 10^17^, 1.4 × 10^21^ and 3.0 × 10^20^ cm^−3^, respectively. And the mobilities are 32.2, 4.1, and 61.9 cm^2^V^−1^s^−1^, respectively.

## Conclusions

In conclusion, we successfully synthesized a series of Mo_1−*x*_W_*x*_Te_2_ single crystals. By means of XRD, Raman spectroscopy, and DFT calculations, we find that by increasing the W composition (*x*), the structure gradually changes from 2H, β to type-II Weyl semimetal Td phase. By changing temperature, the high temperature β-phase of Mo_1−*x*_W_*x*_Te_2_ is evolved to low temperature Td-phase. Accordingly, temperature-dependent and composition-dependent phase diagram of Mo_1−*x*_W_*x*_Te_2_ is proposed. Simultaneously, the electrical property gradually evolves from semiconductor in 2H phase to semimetal β phase then to semimetal Td phase. This work provides a useful map to explore the type-II topological Weyl semimetal phase and temperature/composition-dependent topological phase transition, as well as the corresponding novel physical properties in Mo_1−*x*_W_*x*_Te_2_ compounds.

## Additional Information

**How to cite this article:** Lv, Y.-Y. *et al*. Composition and temperature-dependent phase transition in miscible Mo_1-*x*_W_*x*_Te_2_ single crystals. *Sci. Rep.* **7**, 44587; doi: 10.1038/srep44587 (2017).

**Publisher's note:** Springer Nature remains neutral with regard to jurisdictional claims in published maps and institutional affiliations.

## Supplementary Material

Supplementary Information
