# Supplementary material for: Composition and temperature-dependent phase transition in miscible Mo1−xWxTe2 single crystals
Source: Sci Rep. 2017 Mar 15;7:44587. doi: 10.1038/srep44587 (PMC5353676; doi:10.1038/srep44587)
Supplement: Supplementary Information [file srep44587-s1.doc]

**Supplementary Information of “Composition and temperature-dependent phase transition in miscible Mo_1-_*_x_*W*_x_*Te_2_ single crystals”**

Yang-Yang Lv^1^, Lin Cao^1^, Xiao Li^2^, Bin-Bin Zhang^1^, Kang Wang^2^, Bin Pang^1^, Ligang Ma^2^, Dajun Lin^1^, Shu-Hua Yao^1*^, Jian Zhou^1*^, Y. B. Chen^2*^, Song-Tao Dong^3^, Wenchao Liu^1,4^, Ming-Hui Lu^1^, Yulin Chen^5,6^ & Yan-Feng Chen^1,7^

^1^National Laboratory of Solid State Microstructures & Department of Materials Science and Engineering, Nanjing University, Nanjing 210093 China

^2^National Laboratory of Solid State Microstructures & Department of Physics, Nanjing University, Nanjing 210093 China

^3^Institute of materials Science and Engineering, Jiangsu University of Science and Technology, Zhenjiang 212003 China

^4^Institute of Advanced Materials (IAM) & Jiangsu National Synergetic Innovation Center for Advanced Materials (SICAM), Nanjing Tech University, Nanjing 211800 China

^5^School of Physical Science and Technology, Shanghai Tech University, Shanghai 200031, China.

^6^State Key Laboratory of Low Dimensional Quantum Physics, Collaborative Innovation Center of Quantum Matter and Department of Physics, Tsinghua University, Beijing 100084, China.

^7^Collaborative Innovation Center of Advanced Microstructure, Nanjing University, Nanjing, 210093 China

**1. The lattice constant for MoTe_2_ and WTe_2_ of 2H, β and Td phase**

**Table I**. Calculated and reported lattice (in the unit of Å) constant for MoTe_2_ and WTe_2_ of 2H, β and Td phase.

| Structure | Calculated lattice constants | Reported lattice constants ^[1]^ |
| --- | --- | --- |
| 2H-MoTe_2_ | a=b=3.530  c=14.068 | a=b=3.519  c=13.97 |
| β-MoTe_2_ | a=6.336  b=3.492  c=13.873  β=93^o^55’ | a=6.330  b=3.469  c=13.86  β=93^o^55’ |
| Td-MoTe_2_ | a=6.332  b=3.493  c=13.849 | a=6.33  b=3.469  c=13.83 |
| 2H-WTe_2_ | a=b=3.530  c=14.112 | a=b=3.600  c=14.18 |
| β-WTe_2_ | a=6.285  b=3.491  c=14.238  β=94^o^3’ | N.A. |
| Td-WTe_2_ | a=6.280  b=3.494  c=14.214 | a=6.282  b=3.496  c=14.07 |

**2. The composition determinations of Mo_1-_*_x_*W*_x_*Te_2_ single crystals**

In the EDS measurements, we have collected the data in more than five different areas and taken the average value. During the quantitative calculations, the scattering cross sections of W, Mo and Te are calibrated by stoichometric WTe_2_ and MoTe_2_ powder and were taken in the all data analysis^[1]^. All the EDS spectra of the as-grown Mo_1-_*_x_*W*_x_*Te_2_ and β-MoTe_2_ crystals are depicted in Fig. S1. The elemental compositions of all the crystal samples are shown in Table II. The ZAF factors of Mo-*L*, W-*L* and Te-*L* peaks are summarized in Table III.


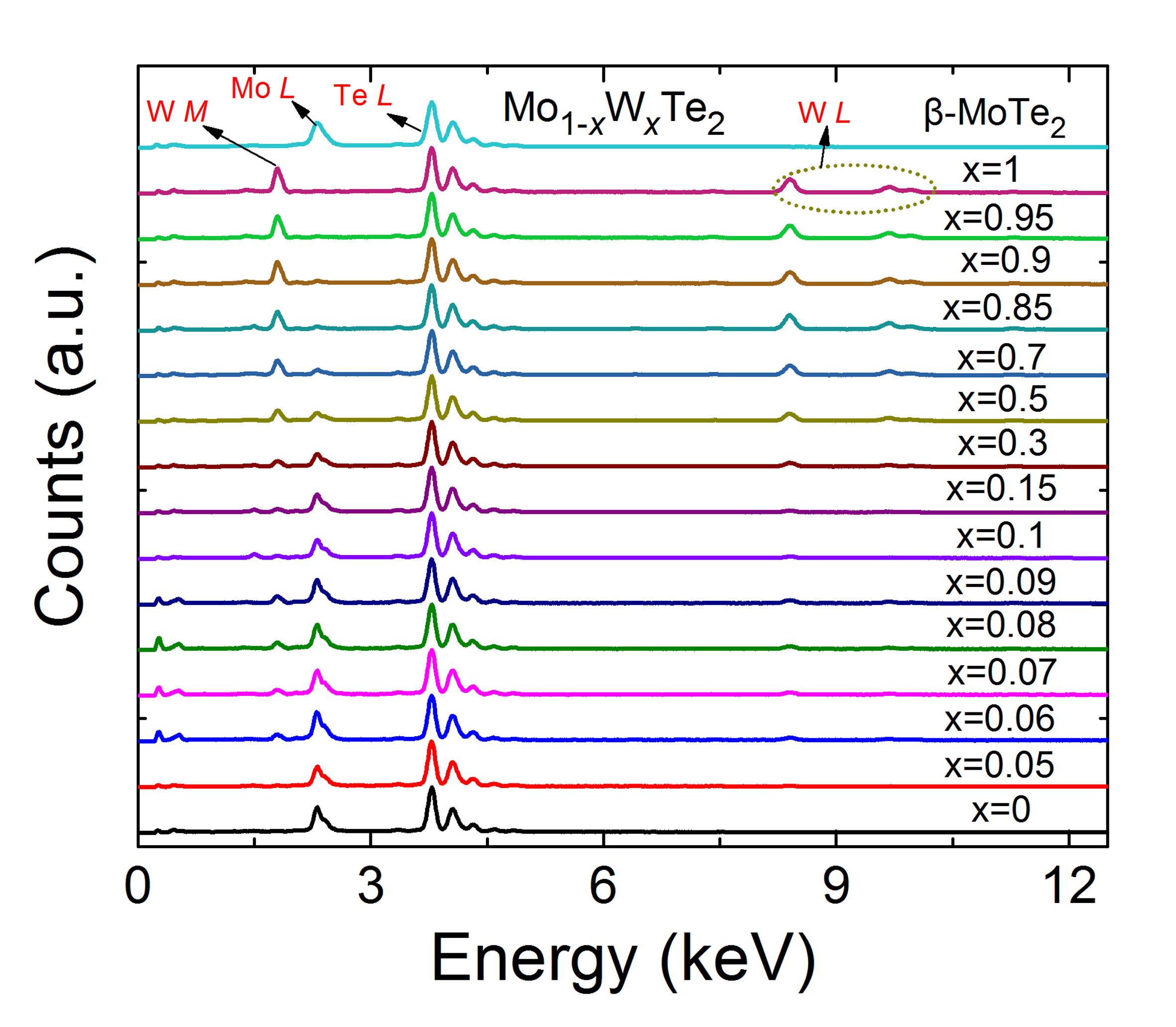


**Figure S1**. EDS spectra of the as-grown Mo_1-_*_x_*W*_x_*Te_2_ and β-MoTe_2_ crystals

**Table II**. The EDS experiment results of Mo_1-_*_x_*W*_x_*Te_2_ and β-MoTe_2_ crystals.

| *x* | Mo (At%) | W (At%) | Te (At%) |
| --- | --- | --- | --- |
| β-MoTe_2_ | 33.12 | 0 | 66.88 |
| 0 | 33.41 | 0 | 66.59 |
| 0.05 | 31.79 | 1.59 | 66.62 |
| 0.06 | 31.62 | 1.96 | 66.42 |
| 0.07 | 31.06 | 2.35 | 66.59 |
| 0.08 | 30.78 | 2.67 | 66.55 |
| 0.09 | 30.77 | 3.05 | 66.18 |
| 0.1 | 30.39 | 3.47 | 66.14 |
| 0.15 | 28.54 | 5.03 | 66.43 |
| 0.3 | 23.67 | 9.89 | 66.44 |
| 0.5 | 16.73 | 17.02 | 66.25 |
| 0.7 | 9.98 | 23.60 | 66.42 |
| 0.85 | 5.11 | 28.62 | 66.27 |
| 0.9 | 3.37 | 30.04 | 66.60 |
| 0.95 | 1.60 | 32.79 | 65.61 |
| 1 | 0 | 33.61 | 66.39 |

**Table III**. The cross section used in EDS analysis of Mo_1-x_W_x_Te_2_.

|  | Z | A | F |
| --- | --- | --- | --- |
| Mo-*L* | 1.0573 | 0.5404 | 1.0117 |
| Te-*L* | 0.9881 | 0.8072 | 1.0000 |
| W-*L* | 0.9702 | 0.9505 | 1.0000 |

**3. The *c*-axis lattice parameter *d_c_* of Mo_1-_*_x_*W*_x_*Te_2_ single crystals**

To show the effect of Mo substituted and non-stoichiometric on the lattice constant clearly, we plot the *c*-axis lattice parameter *d_c_* for all Mo_1-_*_x_*W*_x_*Te_2_ single crystals in Fig. S2. It can be seen clearly that there are three continuous change regimes (*x*=0~0.07, 0.10~0.50, and 0.70~1, respectively), which implies there may be three different phases. This may be due to the different lattice parameter *c* of 2H-MoTe_2_, β-MoTe_2_ and Td-WTe_2_ (13.97, 13.86, and 14.07 Å, respectively)^[2]^. But, by careful inspection, we find that from *x*=0.08 to 0.10 and from *x*=0.50 to 0.70, the *d_c_* gradually decreases with increasing *x* (W composition). So it is reasonable to conclude that the phase transitions occur from *x*=0.08 to 0.1 and from *x*=0.50 to 0.70. Crystal structures of Mo_1-_*_x_*W*_x_*Te_2_ compounds change from 2H, β to Td phase with increasing *x*.


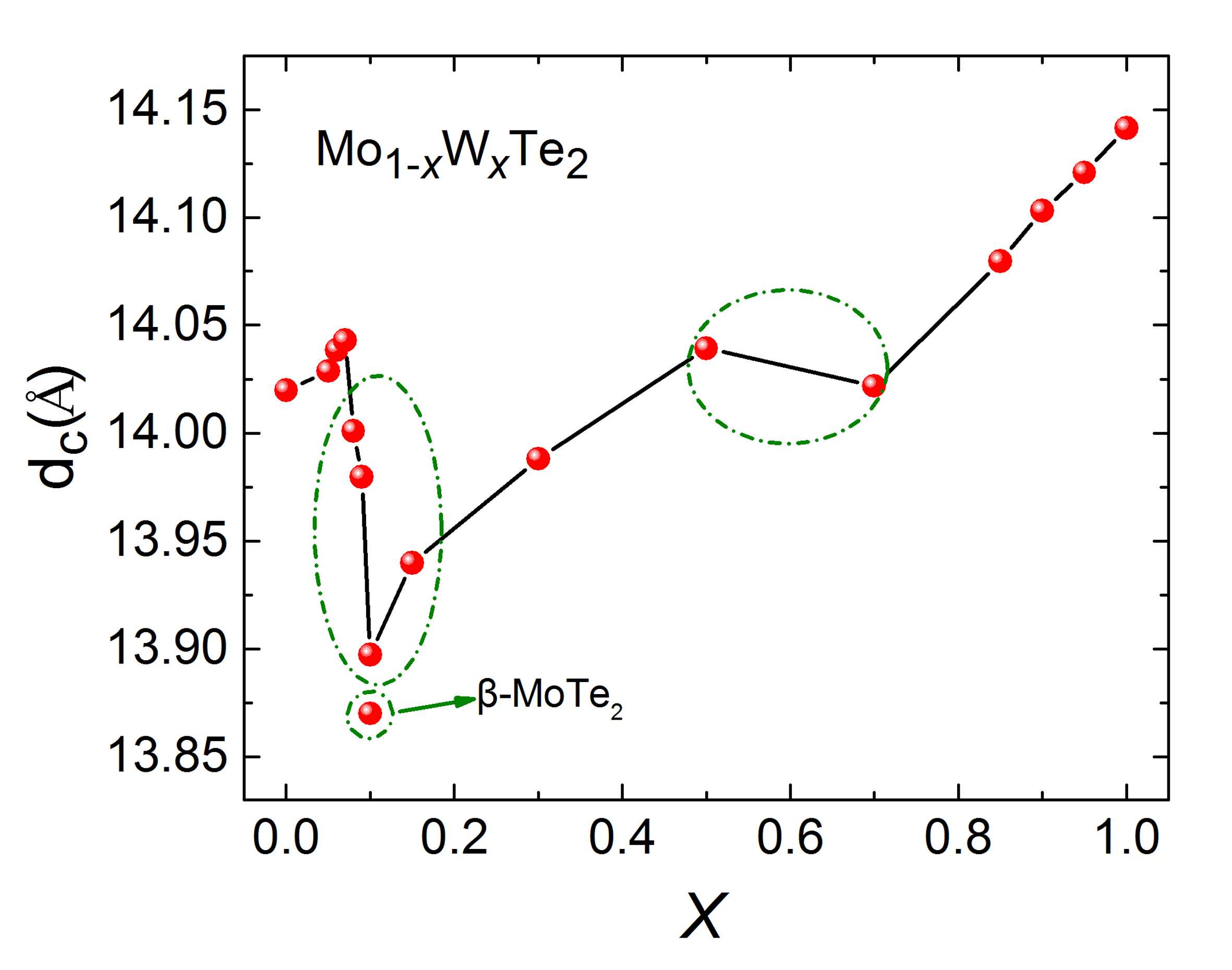


**Figure S2**. The *c*-axis lattice parameter *d_c_* as a function of *x* for Mo_1-_*_x_*W*_x_*Te_2_ system and β-MoTe_2_, respectively.

**4. The calculated and experimental Raman spectra for MoTe_2_ and WTe_2_ of 2H, β and Td phase**


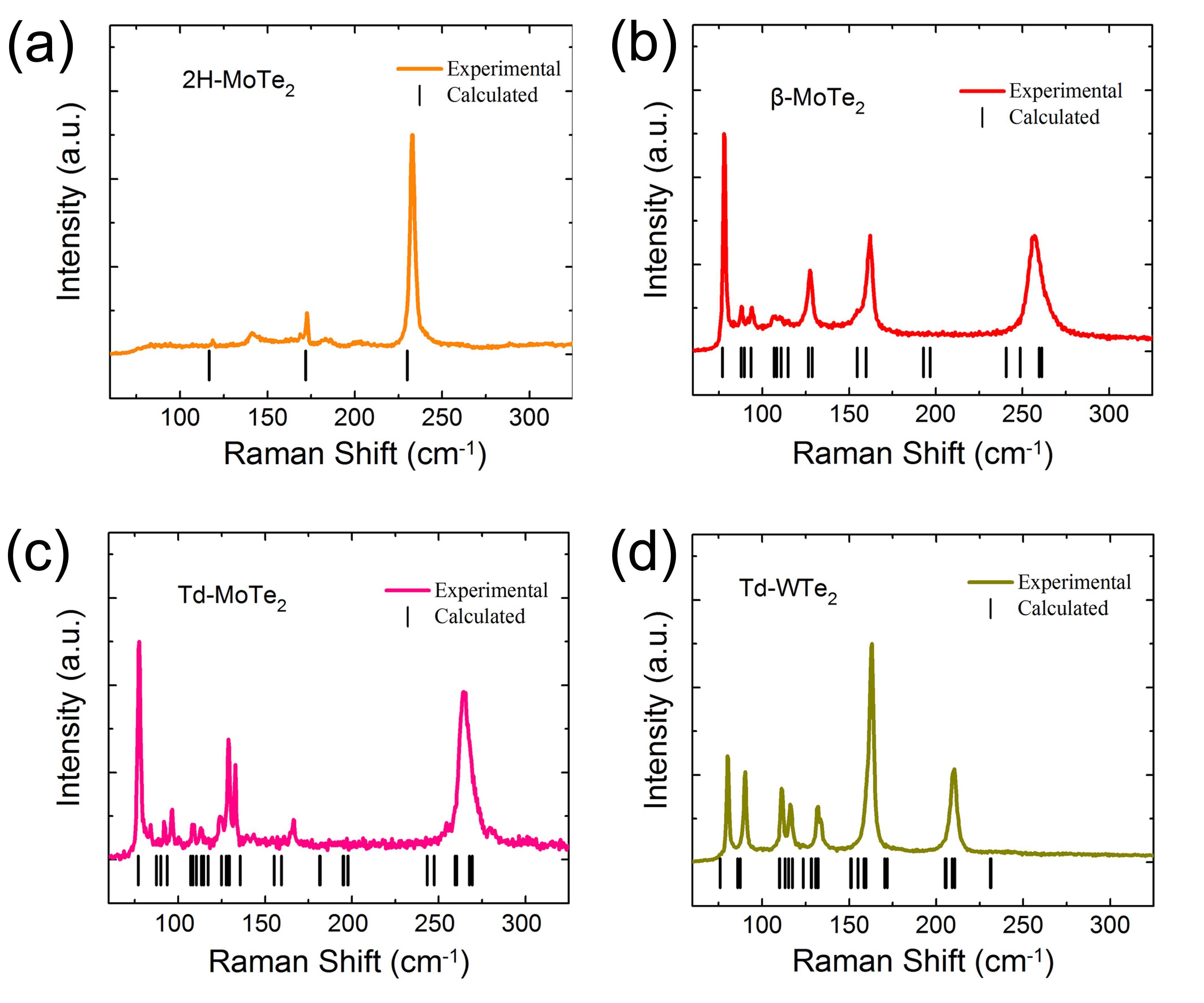


**Figure S3**. The calculated and experimental Raman spectra for MoTe_2_ and WTe_2_ of 2H, β and Td phase: (a) 2H-MoTe_2_; (b) β-MoTe_2_; (c) Td-MoTe_2_; (d) Td-WTe_2_.

**5. The Raman spectra of Mo_1-_*_x_*W*_x_*Te_2_ (*x*=0.3, 0.5 and 0.7) single crystals**


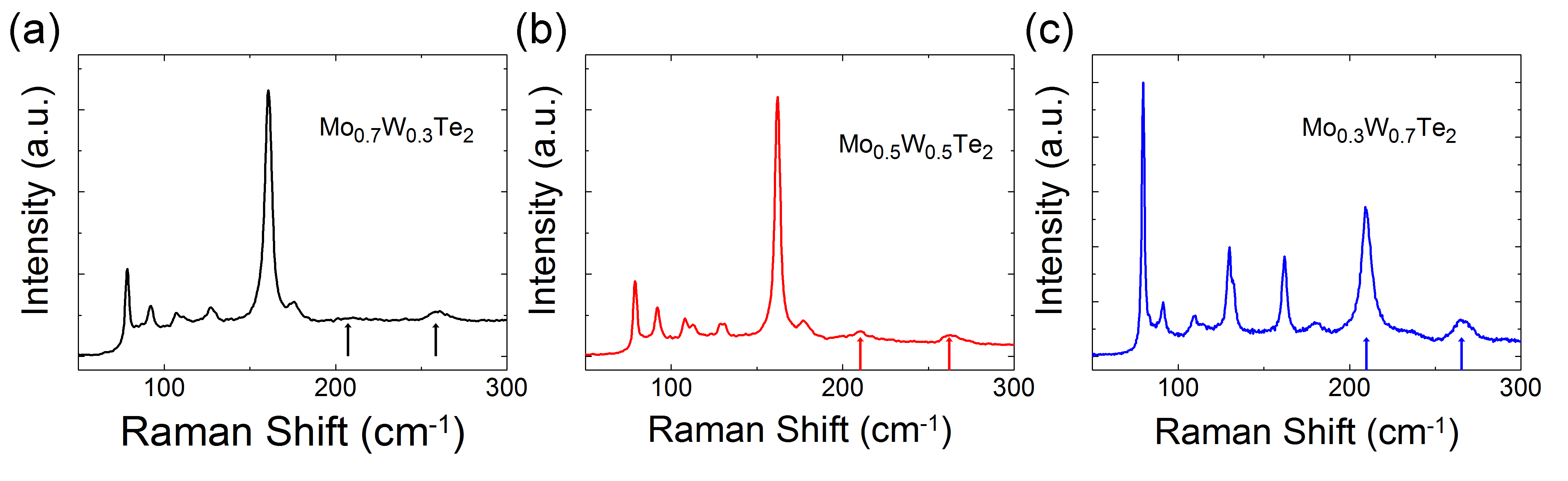


**Figure S4**. The Raman spectra of Mo_0.7_W_0.3_Te_2_ (a), Mo_0.5_W_0.5_Te_2_ (b) and Mo_0.3_W_0.7_Te_2_ (c) crystals measured at room temperature.

**6. The calculations of carrier concentrations and carrier mobilities for Mo_1-_*_x_*W*_x_*Te_2_ and β-MoTe_2_ single crystals**

The Hall resistivities of Mo_1-_*_x_*W*_x_*Te_2_ single crystals were measured by standard four-probe technique. According to the principle of Hall-effect measurements^[3]^, the carrier concentrations can be expressed as

(1),

and the carrier mobilities can be expressed as

(2)

where *n* is carrier concentration, *R_H_* is Hall coefficient, *d* is the thickness of the sample, *B* is the applied magnetic field, *e* is the charge of the electron, *ρ_xy_* is Hall resistivity, *μ* is carrier mobility, *ρ_xx_* is the electrical resistivity, respectively.

**References**

[1] Shindo, D. & Oikawa, T. *Analytical Electron Microscopy for Materials Science* (Springer-Verlag, Tokyo, 2002).

[2] Dawson, W. G. & Bullett, D. W. Electronic structure and crystallography of MoTe_2_ and WTe_2_. *J. Phys. C: Solid State Phys.* 20, 6159-6174 (1987).

[3] Putley, E. H. The hall effect and its applications. *Contemp. Phys.*, 16, 101-126 (1975).
